# May clinical neurophysiology help to predict the recovery of neurological early rehabilitation patients?

**DOI:** 10.1186/s12883-015-0496-9

**Published:** 2015-11-21

**Authors:** Jens D. Rollnik

**Affiliations:** Institute for Neurorehabilitation Research (“InFo”), BDH Clinic Hessisch Oldendorf, Hannover Medical School (MHH), Greitstr. 18-28, 31840 Hessisch Oldendorf, Germany

**Keywords:** Clinical neurophysiology, AEP, VEP, SEP, EEG, Outcome, Prognosis, Early rehabilitation

## Abstract

**Background:**

So far, the role of clinical neurophysiology in the prediction of outcome from neurological and neurosurgical early rehabilitation is unclear.

**Methods:**

Clinical and neurophysiological data of a large sample of 803 early rehabilitation cases of the BDH-Clinic Hessisch Oldendorf in Northern Germany have been carefully reviewed. Most patients (43.5 %) were transferred to rehabilitation after stroke, mean age was 66.6 (15.5) years. Median somatosensory (SEP), auditory (AEP) and visual evoked potentials (VEP) along with EEG recordings took place within the first two weeks after admission. Length of stay (LOS) in early rehabilitation was 38.3 (37.2) days.

**Results:**

Absence of SEP on one or both sides was associated with poor outcome, *χ*2 = 12.98 (*p* = 0.005); only 12.5 % had a good outcome (defined as Barthel index, BI ≥50) when SEP were missing on both sides. In AEP, significantly longer bilateral latencies III were observed in the poor outcome group (*p* < 0.05). Flash VEP showed that patients in the poor outcome group had a significantly longer latency III on both sides (*p* < 0.05). The longer latency III, the smaller BI changes (BI discharge minus admission) were observed (latency III right *r* = −0.145, *p* < 0.01; left *r* = −0.206, *p* < 0.001). While about half of the patients with alpha EEG activity belonged to the good outcome group (80/159, 50.3 %), only 39/125 (31.2 %) with theta and 5/41 (12.2 %) with delta rhythm had a favourable outcome, *χ*2 = 24.2, *p* < 0.001.

**Conclusions:**

Results from this study suggest that loss of median SEP, prolongation of wave III in AEP and flash-VEP as well as theta or delta rhythms in EEG are associated with poor outcome from neurological early rehabilitation. Further studies on this topic are strongly encouraged.

## Background

Patients entering neurological and neurosurgical early rehabilitation are severely impaired. Morbidity is high [1] and they are suffering from disorders of consciousness [2, 3]. Early rehabilitation patients are dependent on nursing and may be colonized with multi-drug resistant germs [4, 5]. Frequently, their outcome is poor, but it is quite difficult to predict outcome accurately [3].

The role of clinical neurophysiology, in particular electroencephalography (EEG) and evoked potentials (EP) in predicting outcome of these patients is still unclear. Only a few studies are available on long-term rehabilitation results and clinical neurophysiology. The question is whether clinical neurophysiological techniques may help to distinguish between patients who benefit from neurological early rehabilitation and such who don’t. In contrast to imaging techniques, neurophysiological measurements are easy to perform, cheap, safe and available in most rehabilitation facilities.

Most studies focus on rehabilitation outcome of stroke patients (Table 1) [6–16]. With respect to SEP, an absence or amplitude reduction of cortical responses seems to be associated with a poor long-term outcome after stroke [6, 7, 10]. Like with SEP, absence of MEP may indicate poor recovery from stroke [10, 16]. VEP have also been studied, suggesting that left-right asymmetry may be associated with functional outcome [9]. Presence of delta and theta activity in EEG predicted unfavorable outcome one year after stroke [8].

**Table 1:** Studies on long-term outcome of stroke patients involving clinical neurophysiological data

Study	*n*	Methods	Results
Zeman & Yiannikas 1989 [6]	35	Median SEP	Abnormal SEP (absence or amplitude reduction or prolonged latency N20) were associated with poor outcome (lower BI) after a mean LOS of 51 days
Kovala 1991 [7]	35	Median and tibial SEP	Tibial SEP: correlation between amplitude abnormalities (absence or attenuation) and occupational outcome after 1 y
			Median SEP: absence of N60 predicted poor outcome
Cillessen et al. 1994 [8]	55	EEG in the acute stage	EEG (presence of delta and theta activity) predicted poor outcome after 1 year
Ring, Bar & Abboud, 1999 [9]	4	VEP	Correlation between left-right asymmetry of VEP and functional outcome after a mean of 137 days inpatient rehabilitation
Feys et al. 2000 [10]	64	Median SEP and upper limb MEP	Absence of SEP and MEP indicated poor outcome 2 months after stroke
Steube, Wiethölter & Correll 2001 [11]	100	Lower limb MEP more than 4 weeks after stroke	Preserved MEP predicted good outcome (independence in walking) after 8 weeks of inpatient rehabilitation
Tzvetanov & Rousseff 2003 [12];	94	Median and tibial SEP	N20-P25 amplitude is of some predictive value (1 y outcome), but MRC is stronger predictor of functional outcome, P40 amplitude correlated with BI (*r* = 0.63) after 3 months
Tzvetanov, Rousseff, & Milanov 2003 [13];			
Tsetanov, Rousseff & Atanassova 2005 [14]			
Al-Rawi, Hamdan & Abdul-Muttalib 2009 [15]	22	Median SEP	Correlation between SEP (N20 latency and amplitude) and 3-month outcome (BI, MRC)
Pizzi et al. 2009 [16]	52	Upper limb MEP	In patients with severe paresis (MRC <2), absence of MEP were predictive of poor recovery

*SEP* somatosensory evoked potentials, *EEG* electroencephalography, *MEP* motor evoked potentials, *VEP* visual evoked potentials, *BI* Barthel index, *MRC* Medical Research Council (severity of paresis)

Some studies on long-term outcome of traumatic brain injury (TBI) patients are available (Table 2) [17–23]. While AEP may be of some prognostic value in this group of patients [17, 18], most studies focus on SEP. Like with stroke, TBI patients with a loss of cortical SEP-responses seem to have a poor outcome [22].

**Table 2:** Studies on long-term outcome of traumatic brain injury (TBI) patients involving clinical neurophysiological data

Study	*n*	Methods	Results
Mackey-Hargadine & Hall 1985 [17]	114	AEP	Significant correlation between AEP, pupil reactivity and outcome in a 24 month period
Shin et al. 1989 [18]	29	AEP and SEP	SEP helped to improve prediction of outcome 12 months after TBI
Thatcher et al. 1991 [19]	162	EEG and AEP	Combination of EEG and GCS bet predicted outcome 12 months after TBI
Mazzini et al. 1999 [20]	27	SEP and MEP from upper and lower limbs	Clinical and functional outcome was strongly correlated with abnormalities from tibial SEP, 6 and 12 months after TBI
Özbudak-Demir et al. 1999 [21]	26	Median and tibial SEP	Normal SEP latencies were associated with better outcome, more than 8 months after TBI
Lew et al. 2003 [22]	22	Median SEP	Bilateral absence of SEP was strongly predictive of worst outcome (death or vegetative state), 6 months after TBI
Houlden et al. 2010 [23]	81	Median SEP	SEP within 7 days after TBI correlate with 1 y outcome

*TBI* traumatic brain injury, *SEP* somatosensory evoked potentials, *AEP* auditory evoked potentials, *EEG* electroencephalography, *MEP* motor evoked potentials

There are also studies on long-term outcome of disorders of consciousness (DOC) patients, in particular after hypoxic brain damage (Table 3) [3, 24–28]. Results on the question whether SEP or AEP may be used to predict recovery are controversial [24–27]. EEG could be of some value [28]. Latest results from our group suggest that prolongation of wave III in flash VEP, theta or delta rhythm in EEG, and N20/P25 amplitude reduction in median SEP may be associated with poor outcome of hypoxic brain damage patients undergoing neurological early rehabilitation [3].

**Table 3:** Studies on long-term outcome of DOC (disorders of consciousness) patients due to hypoxic or severe brain damage of different origin involving clinical neurophysiological data

Study	*n*	Methods	Results
Zeitlhofer et al. 1991 [24]	22	AEP and SEP	Evoked potentials had no value for the prognosis of “apallic” patients
Goldberg & Karazim 1998 [25]	33	AEP and SEP	AEP and SEP predicted outcome of MCS patients
Howell et al. 2013 [26]	113	SEP	SEP did not predict outcome of hypoxic brain damage survivors
Schorl, Valerius-Kukula & Kemmer 2014 [27]	28	Median SEP	Bliateral loss of SEP did not exclude recovery from severe brain damage
Bagnato et al. 2015 [28]	101	EEG	Reduced EEG amplitudes and delta frequencies were associated with bad clinical outcome (UWS, MCS)
Heinz & Rollnik 2015 [3]	93	EEG, flash VEP, median SEP	Prolongation of wave III (flash VEP), theta or delta EEG rhythm and N20/P25-amplitude reduction (SEP) predicted poor outcome (BI <50)

*SEP* somatosensory evoked potentials, *AEP* auditory evoked potentials, *EEG* electroencephalography, *VEP* visual evoked potentials, *BI* Barthel index, *UWS* unresponsive wakefulness syndrome, *MCS* minimally conscious state

The present study tried to figure out whether clinical neurophysiology may help to improve outcome prediction of a large cohort of neurological and neurosurgical early rehabilitation patients.

## Methods

### Patients

Medical records of 803 patients (376 female, 436 male) of the BDH-Clinic Hessisch Oldendorf, a large neurological and neurosurgical early rehabilitation facility located in Northern Germany, have been analyzed. Patients were admitted in 2010. Main diagnoses are presented in Table 4. The main diagnosis was defined according to the G-DRG- (German Diagnosis Related Groups) system as the disease justifying referral for early rehabilitation. When stroke patients also suffered from a peripheral disorder like diabetic polyneuropathy, for instance, stroke was defined as main diagnosis because it necessitated rehabilitation treatment.

**Table 4:** Main diagnoses

	Number	Percent
Stroke	349	43.5
Intracerebral bleeding	107	13.3
Subarachnoidal bleeding	65	8.1
Hypoxic brain damage	37	3.4
Polyneuropathy, GBS	24	3.0
Brain tumor	23	2.9
Traumatic brain injury	21	2.6
Spinal trauma	13	1.6
Meningitis, encephalitis	10	1.2
Other main diagnosis	154	19.2
Sum	803	100

Most patients underwent early rehabilitation after stroke (*n* = 349, 43.5 %), 34.0 (208.6) days after disease onset. Mean age was 66.6 (15.5) years. Length of stay (LOS) in early rehabilitation was 38.3 (37.2) days, LOS of the whole rehabilitation 58.5 (52.4) days. Discharge placement: 52.2 % (419/803) underwent subsequent rehabilitation or went home, 31.5 % (253/803) were discharged to a nursing home, 11.3 % (91/803) needed acute-care hospital treatment and 4.7 % (38/803) died. One patient was discharged against medical advice and another one was transferred to a hospice.

### Clinical scales and assessments

On admission and at discharge, Barthel index (BI) [29] and Early Rehabilitation Index (ERI) [30] have been obtained. In line with previous studies, poor outcome was defined as a BI <50 points [3]. In addition, CRS [31], GCS [32] and Early Functional Abilities scale [33] have been analyzed.

### Clinical neurophysiology

Electroencephalography (EEG), auditory evoked potentials (AEP), visual evoked potentials (VEP), somatosensory evoked potentials (SEP) of the median nerve were recorded usually within the first two weeks after admission. EEG was done using the international 10/20 system (Neurofax EEG 9000, Nihon Kohden Europe, Rosbach, Germany). Surface electrodes were used for evoked potentials (Nicolet Viking Select, Natus Medical, Middleton, WI, USA). VEPs were recorded with flashing light-emitting diodes (flash VEP, stimulation frequency 1.3Hz). Latencies and amplitudes of wave I-III were examined according to the guidelines of the American Clinical Neurophysiology Society [34]. Further, AEP latencies I-V and N20/P25 latencies and amplitudes of median nerve SEPs were analyzed. Neurophysiological examinations were performed by an experienced team of only four paramedics working in this field for many years.

### Ethics

This is a retrospective data analysis, only (no intervention). Local ethics committee of the BDH-Clinic Hessisch Oldendorf gave approval to use facility’s data. Patient records/information were anonymized and de-identified prior to analysis. No written informed consent for participation was obtained (retrospective data analysis, no intervention).

### Statistics

For statistical analyses, SPSS™ 21.0 software package (SPSS Inc, Chicago, USA) was used. In the results section, mean values and standard deviations (in brackets) are displayed. In parametric (t-tests for independent samples and analysis of variance) as well as non-parametric tests (*χ*^2^-tests), differences were regarded as significant with *p* < 0.05. In addition, bivariate Pearson correlations were computed.

## Results

Poor outcome patients were significantly older, had more co-diagnoses, a longer LOS, lower BI, ERI, GCS and CRS on admission (Table 5).

**Table 5:** Characteristics of neurological early rehabilitation patients with good and poor outcome

	good outcome	poor outcome	p-value*
Age [years]	62.4 (15.7)	69.0 (14.7)	<0.001
LOS – neurological early rehabilitation [days]	27.8 (38.0)	46.1 (35.3)	<0.001
Number of co-diagnoses [n]	13.0 (5.4)	16.6 (5.5)	<0.001
Barthel Index (BI) on admission [0 to 100]	33.0 (28.5)	14.4 (8.2)	<0.001
Barthel index at discharge [0 to 100]	76.5 (16.6)	20.7 (10.7)	<0.001
Delta BI (discharge minus admission)	43.5 (25.7)	6.3 (9.9)	<0.001
Early Rehabilitation Index (ERI) on admission [−325 to 0]	−46.5 (49.3)	−59.1 (53.6)	0.001
ERI at discharge [−325 to 0]	−14.8 (23.9)	−38.5 (46.2)	<0.001
Coma Remission Scale (CRS) [0 to 24]	15.9 (6.3)	10.1 (6.4)	<0.001
Glasgow Coma Scale (GCS) on admission [3 to 15]	12.8 (2.9)	10.1 (3.6)	<0.001
Glasgow Coma Scale (GCS) at discharge [3 to 15]	14.4 (0.5)	11.9 (3.6)	n.s.

*t-tests for independent samples, *n.s.* not significant (*p* > 0.05)

### Imaging data

Computed tomography (CT) and/or magnetic resonance imaging (MRI) of the skull was available in about 2/3 of cases (Table 6). Most frequent lesion sites were temporal, parietal and frontal lobes.

**Table 6:** Imaging results (lesion site)

Brain region	Left	Right	Bilateral	Sum
Temporal lobe	106 (13.2 %)	115 (14.3 %)	39 (4.9 %)	260 (32.4 %)
Parietal lobe	76 (9.5 %)	127 (15.8 %)	42 (5.2 %)	245 (30.5 %)
Frontal lobe	41 (5.1 %)	57 (7.1 %)	53 (6.6 %)	151 (18.8 %)
Occipital lobe	25 (3.1 %)	29 (3.6 %)	24 (3.0 %)	78 (9.7 %)
Brain stem	16 (2.0 %)	17 (2.1 %)	27 (3.4 %)	60 (7.5 %)
Cerebellum	12 (1.5 %)	17 (2.1 %)	14 (1.7 %)	43 (5.3 %)

### Median SEP

Median SEP data was available in 449 cases (55.9 %). Loss of cortical SEP on one or both sides was associated with poor outcome (Table 7), *χ*^2^ = 12.98 (*p* = 0.005). While 153/353 (43.3 %) had a good outcome when SEP were present, only 26.3 % (21/80) belonged to the good outcome group when SEP were absent on one side and 12.5 % (2/16) when SEP were absent on both sides. Neither N20 or P25 latencies, nor SEP amplitudes were different between good and poor outcome group (Table 8). The age of patients with loss of SEP on one or both sides compared to those with no absence of SEP did not differ significantly (*F* = 2.213, *p* > 0.05).

**Table 7:** Absence of median SEP on one or both sides and outcome categories

	Absence of median SEP				Sum
Outcome	*none*	*absence right*	*absence left*	*bilateral absence*	
Poor	200	24	35	14	273
Good	153	9	12	2	176
Sum	353	33	47	16	449

**Table 8:** Data of evoked potentials

Outcome	GOOD		POOR	
	Left	Right	Left	Right
Auditory evoked potentials (AEP)				
Latency I [ms]	1.71 (0.18)	1.70 (0.18)	1.72 (0.20)	1.73 (0.19)
Latency II [ms]	2.82 (0.24)	2.84 (0.27)	2.84 (0.25)	2.83 (0.25)
Latency III [ms]	3.95 (0.25)*	3.95 (0.25)**	4.01 (0.26)*	4.02 (0.27)**
Latency IV [ms]	5.09 (0.32)*	5.09 (0.29)	5.15 (0.33)*	5.12 (0.33)
Latency V [ms]	5.93 (0.32)	5.95 (0.30)	5.95 (0.32)	5.98 (0.32)
Visual evoked potentials (flash VEP)				
Latency I [ms]	52.7 (15.7)	53.2 (14.5)	52.4 (13.7)	52.8 (13.2)
Latency II [ms]	75.7 (17.3)	77.1 (15.9)	78.2 (16.9)	78.6 (16.0)
Latency III [ms]	108.1 (20.7)*	110.2 (20.2)*	115.2 (21.1)*	115.5 (19.6)*
Amplitude I/II [μV]	8.3 (7.9)	8.7 (8.2)	7.9 (6.2)	8.3 (6.2)
Somatosensory evoked potentials (SSEP) of the median nerve				
N20 [ms]	21.0 (1.8)	20.8 (2.5)	21.1 (2.1)	21.1 (1.9)
P25 [ms]	26.1 (2.7)	26.3 (3.2)	25.7 (3.0)	26.2 (3.1)
Amplitude N20/P25 [μV]	3.7 (2.4)	3.9 (3.0)	4.0 (3.7)	3.8 (4.0)

Significant differences between subjects with good and poor outcome are indicated as follows: *p < 0.05, **p < 0.01 (t-tests for independent samples)

### AEP

AEP data was available in 448 cases (55.8 %). Absence of AEP on one side was observed in two cases, bilateral loss of AEP responses in one case, only. All three cases belonged to the poor outcome group, but due to small sample size, *χ*^2^-test did not reveal significant differences. When comparing poor and good outcome groups, it turned out that a significantly longer latency III was observed on both sides in the poor outcome group (*p* < 0.05), Table 8. Latency IV also showed significant differences between the groups, but on the left side, only (*p* < 0.05). Since AEP are closely connected to brain stem function, a sub-analysis focusing on brain stem lesions was done: 60 patients with brainstem lesions (7.5 %) were identified. With intact brain stem, BI at discharge was significantly higher than in patients with uni- or bilateral lesion (*F* = 3.931, *p* = 0.009). Further, there was a small but significant correlation between age and AEP latency III (left: *r* = 0.209, *p* < 0.001; right: *r* = 0.132, *p* < 0.01).

### VEP

Flash VEP data was available in 391 cases (48.7 %). Loss of cortical VEP was detected in six cases on one and in four cases on both sides, only. All bilateral loss cases belonged to the poor outcome group. With unilateral absence, 4/6 (66.6 %) had a poor outcome. Like with AEP, these differences did not reach a level of significance due to small group size. Patients belonging to the poor outcome group had a significantly longer flash VEP latency III on both sides (*p* < 0.05), Table 8. The longer latency III, the smaller BI changes (BI discharge minus admission) could be observed (latency III right *r* = −0.145, *p* < 0.01; left *r* = −0.206, *p* < 0.001). As with AEP, there was a small but significant correlation between age and VEP latency III (left: *r* = 0.166, *p* < 0.01; right: *r* = 0.136, *p* < 0.01).

### EEG

**Fig. 1:**
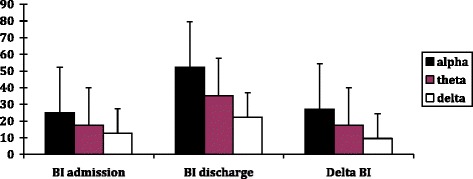
When patients had alpha EEG-activity, BI on admission, at discharge and changes of BI (discharge minus admission) were significantly higher than patients with theta or delta activity (ANOVAs with LSD-tests, *p* < 0.001)

EEG recordings were available in 360 cases (44.8 %). EEG with alpha, theta or delta frequency was included in the analysis, Table 9. While about half of the patients with alpha activity belonged to the good outcome group (80/159, 50.3 %), only 39/125 (31.2 %) with theta activity and 5/41 (12.2 %) with delta rhythm had a favorable outcome, *χ*^2^ = 24.2, *p* < 0.001. In ANOVAs, BI was significantly lower on admission and discharge when patients had theta or delta rhythms compared to alpha activity (*p* < 0.001). BI changes (BI discharge minus admission) were also smaller when patients had delta or theta activity, Fig. 1. The age of patients with alpha, theta or delta activity did not differ significantly (*F* = 1.274, *p* > 0.05).

**Table 9:** EEG activity and outcome

	EEG frequency			
Outcome	*Alpha*	*Theta*	*Delta*	Sum
Poor	79	86	36	201
Good	80	39	5	124
Sum	159	125	41	325

## Discussion

So far, reliable data on the usefulness of neurophysiological measurements in predicting the outcome from neurological and neurosurgical early rehabilitation is lacking. The present study analysed data of a large sample 803 patients. One would opt for clinical neurophysiology as a predictor because it is cheap, safe (no radiation), easy to perform and available in most rehabilitation facilities (in contrast to imaging like CT or MRI).

As with previous studies, present results suggest that outcome of neurological early rehabilitation patients mainly depends on age, morbidity and functional status on admission [3]. However, neurophysiological data may be of some predictive value, in particular median SEP, AEP, flash VEP and EEG.

It turned out that patients with worse outcome had longer AEP III latencies. AEP wave III represents the cochlear nucleus which is located in the pontomedullary junction of the dorsolateral brainstem [35]. It is well known that brainstem lesions are associated with poor neurological outcome and fatality [36]. This finding could be reproduced in this study: Subjects with brain stem lesions on one or both sides showed a worse functional outcome from neurological early rehabilitation. As yet, a prolongation of AEP wave III latency has not been identified as a predictor of poor outcome and is a novel finding. Age, however, correlated significantly with wave III latency. Since age is a well-known predictor of poor outcome in neurological rehabilitation [3], it may partially explain this finding. In addition, it has to be pointed out that even in the normal ageing brain, a delay of evoked potentials, in particular VEP and AEP, may be observed [37, 38].

Another finding of this study was a prolongation of wave III in flash VEP in the poor outcome group. This finding is in line with a previous study from our group which focused on hypoxic brain damage patients [3]. VEP wave III abnormalities might be a neurophysiological correlate of cortical dysfunction [3]. As with AEP, VEP wave III latency also correlated with age. Thus, age might influence AEP wave III, too.

Another finding was that loss of cortical median SEP responses on one or both sides was associated with poor outcome. We know from literature that long-term outcome of stroke patients is also worse with absent SEP [6, 10]. Thus, it may be hypothesized that absence of SEP indicates poor outcome in early rehabilitation patients.

There are a couple of studies focusing on EEG and outcome prediction. As with previous studies [3, 8], theta and delta activity was associated with poor outcome.

There are some limitations to this study. First of all, this was a retrospective data analysis, only. This explains why only a proportion of the sample has been studied with all four neurophysiological examinations (EEG, SEP, AEP, VEP). Secondly, the patients showed a wide heterogeneity. This, however, is a common finding when examining neurological early rehabilitation patients [1]. These patients suffer from a broad specter of neurological and neurosurgical disorders, central as well as peripheral disturbances. In addition, the study employed no control group and confounding medication (e.g. neuroleptics, benzodiazepines) has not been included in the analysis. Sedatives, however, are rarely used in our rehabilitation facility.

Results from this study defy ready summary, but EEG, median SEP, AEP and flash VEP may be of some predictive value in early rehabilitation patients. Further studies are strongly encouraged.

## Conclusion

Results from this study suggest that loss of median nerve SEP, prolongation of wave III in early AEP and flash-VEP, as well as theta or delta rhythms in EEG are associated with poor outcome from neurological early rehabilitation. Clinical neurophysiology may help to improve outcome prediction of neurological and neurosurgical rehabilitation patients. EEG and evoked potentials are widely-used, cheap, easy to perform and non-invasive.

## Abbreviations

AEP: Auditory evoked potentials
BI: Barthel index
CRS: Coma remission scale
CT: Computed tomography
DOC: Disorders of consciousness
EEG: Electroencephalography
EFA: Early functional abilities
EP: Evoked potentials
ERI: Early rehabilitation index
GCS: Glasgow coma scale
LOS: Length of stay
MEP: Motor evoked potentials
MRI: Magnetic resonance imaging
SEP: Somatosensory evoked potentials
TBI: Traumatic brain injury
VEP: Visual evoked potentials
